# Gremlin-1 in pregnancy and postpartum: relation to the fatty liver index, markers of bone health, glucose metabolism and gestational diabetes mellitus status

**DOI:** 10.1007/s00592-023-02151-7

**Published:** 2023-07-30

**Authors:** Carola Deischinger, Magdalena Bastian, Karoline Leitner, Dagmar Bancher-Todesca, Herbert Kiss, Sabina Baumgartner-Parzer, Alexandra Kautzky-Willer, Jürgen Harreiter

**Affiliations:** 1 Gender Medicine Unit, Clinical Division of Endocrinology and Metabolism, Department of Internal Medicine III, Medical University of Vienna, Waehringer Guertel 18–20, 1090 Vienna, Austria; 2 Division of Fetomaternal Medicine, Department of Obstetrics and Gynaecology, Medical University of Vienna, Waehringer Guertel 18–20, 1090 Vienna, Austria

**Keywords:** Pregnancy, Gestational diabetes mellitus, Insulin resistance, Gremlin-1, Obesity, Overweight, Fatty liver, Non-alcoholic fatty liver disease, Bone metabolism

## Abstract

**Introduction:**

Gremlin-1 is a peptide that functions as an antagonist to bone morphogenic proteins and is overexpressed in obesity and type 2 diabetes mellitus. Gremlin-1 has not yet been investigated in pregnancy, pregnancy-related insulin resistance or gestational diabetes mellitus (GDM).

**Patients and methods:**

Gremlin-1 levels were measured throughout the pregnancy of 58 women at high risk for GDM at the Medical University of Vienna. Furthermore, an oral glucose tolerance test, fasting insulin, fasting glucose, sex hormones, blood lipids, liver and renal parameters, and markers of bone development were evaluated at two points during pregnancy (< 20 weeks of gestation (GW), GW 24–28) and 12–14 weeks postpartum.

**Results:**

Gremlin-1 levels decreased from < 20 GW (mean = 9.2 pg/ml, SD = 8.4 pg/ml) to GW 24–28 (mean = 6.7 pg/ml, SD = 5.7 pg/ml, *p* = 0.033) and increased again postpartum, albeit not significantly (mean = 10.7 pg/ml, SD = 13.1 pg/ml, *p* = 0.339). During pregnancy, Gremlin-1 levels correlated negatively with osteocalcin and procollagen type I aminoterminal propeptide (P1NP), markers of bone health. Concerning glucose metabolism, Gremlin-1 levels were inversely related to the Insulinogenic Index at GW < 20. However, Gremlin-1 levels were not significantly different between women with normal glucose tolerance and GDM during pregnancy. Postpartum, Gremlin-1 was associated with the fatty liver index, osteocalcin levels, diastolic blood pressure and weight.

**Conclusion:**

Gremlin-1 levels decreased significantly during pregnancy. The biomarker is not related to GDM status, but correlates negatively with the Insulinogenic Index, an index related to beta cell function.

*Trial Registry Number* ACTRN12616000924459.

## Introduction

Gremlin-1 is a peptide belonging to the differential screening-selected gene in neuroblastoma (DAN) and Cerberus protein family and functions as antagonists to bone morphogenic proteins (BMP). Gremlins specifically bind to BMP-2, -4 and -7 [1, 2]. In obesity, subcutaneous adipose tissue cells become BMP-4 resistant due to the secretion of the BMP-4 antagonist Gremlin-1, which then prevents beige/browning of subcutaneous adipose tissue (SAT) [3]. Gremlin-1 has been found to be involved in adipose tissue dysfunction and insulin resistance, antagonizes insulin action and is overexpressed in type 2 diabetes, obesity, non-alcoholic fatty liver disease (NAFLD) and in polycystic ovarian syndrome (PCOS) [4]. Besides metabolic disorders, Gremlin-1 has been found to be increased in chronic fibrotic diseases of the heart following myocardial ischemia [5], the kidney [6] and lung [7], and involved in initiation and progression of various types of cancer [8].

Concerning metabolic diseases, Gremlin-1 is likely involved in impaired subcutaneous adipose tissue (SAT) adipogenesis in hypertrophic obesity. Subcutaneous adipose tissue) is limited in its ability to expand by recruiting and differentiating available precursor cells. When impaired, this can lead to a hypertrophic expansion of adipose cells which is associated with increased inflammation, insulin resistance and lipid accumulation in liver and skeletal muscle tissue due to a reduced ability to store excess fat [9]. A genetic predisposition for type 2 diabetes mellitus is associated with an impaired SAT adipogenesis, which in turn leads to hypertrophic obesity also in nonobese subjects. Previous studies have demonstrated that increased adipose tissue and circulating levels of BMP4 can promote the browning of SAT and, thus, counteract obesity by reducing body weight and ameliorating insulin sensitivity [10, 11], However, while BMP-4 levels are increased in obesity [3, 12], beige/brown adipose cell markers are reduced. Hammarstedt et al. have concluded that, despite higher levels of BMP-4, the activity might be impaired after binding to the antagonist Gremlin-1 and white to beige/brown adipocyte conversion hindered [3, 12]. Antagonizing Gremlin-1 has been hypothesized to be a possibility to further the beige/browning process of SAT, which might lead to a reduced body weight and better insulin sensitivity [3].

Increasing insulin resistance is a normal part of pregnancy as levels of insulin resistance and insulin production increase to divert glucose to the placenta and, consequently, to the foetus [13, 14]. However, if the mother's body does not adequately adapt to these changes, gestational diabetes mellitus (GDM) can develop [15]. GDM is a form of hyperglycaemia in pregnancy which affect about 2–6% of all pregnant women in Europe [16]. Although hyperglycaemia in GDM is usually self-limited to pregnancy, the risk for mothers to develop diabetes mellitus at a later stage in life is 3.5-fold increased [17, 18]. Complications of GDM during pregnancy, childbirth and postpartum affect both mother and child [17, 18]. Risks include preeclampsia, caesarean delivery, foetal macrosomia, intrauterine foetal demise and neonatal hypoglycaemia [17, 18].

As Gremlin-1 has been described to be connected to adipose tissue, type 2 diabetes mellitus and parameters of insulin resistance [4], we aimed at investigating Gremlin-1 in pregnancy, postpartum and the development of GDM. To the best of our knowledge, Gremlin-1 has not yet been studied in the context of pregnancy.

## Materials and methods

### Study participants, methods and design

58 pregnant women were included in a prospective longitudinal study (Ethics Committee of the Medical University of Vienna, EK numbers 2022/2012, 1337/2016) conducted at the Medical University of Vienna between 2013 and 2020. All participants gave their informed consent prior to their inclusion in the study [19]. Women could participate if they fulfilled the inclusion criteria of a singleton pregnancy, gestational age < 20 weeks gestation, age ≥ 18 years and a risk factor for developing GDM warranting an oral glucose tolerance test (OGTT) in early pregnancy according to the Austrian diabetes society guidelines [20], e.g., overweight or obese before pregnancy. Exclusion criteria included pre-existing diabetes mellitus, major active medical disorders, significant psychiatric disorders requiring antipsychotic medication or inability to give consent or follow instructions related to the studies due to a language barrier, women > 20 weeks of gestation (GW), no risk factor for GDM, twins or triplets or already attended an OGTT. As the Medical University of Vienna is a tertiary healthcare centre, we are mostly taking care of high-risk pregnancies, thus, an above average number of participants were overweight or obese and/or developed GDM in our cohort. GDM status was assessed according to the WHO 2013 guidelines [21]. Assessments at each visit included an OGTT, laboratory analyses (including the biomarker Gremlin-1; for details see the chapter “Assay”) and the measurement of blood pressure, weight, height, waist, and hip circumference. The methodology is described in more details in previously published papers on a similar cohort [22–24]. Laboratory measurements included haemoglobin A1c (HbA1c), fasting insulin, fasting glucose, triglycerides (TG), cholesterol, low density lipoprotein cholesterol (LDL-C), high-density lipoprotein cholesterol (HDL-C), oestradiol, testosterone, progesterone, osteocalcin, procollagen type 1 N-terminal propeptide (P1NP), creatinine, alkaline phosphatase (AP), gamma-glutamyl transferase (GGT), aspartate aminotransferase (AST), alanine aminotransferase (ALT) and calcium levels. The fatty liver index (FLI) was calculated with the following formula [25].

$${\text{FLI}} = (e^{{0.953*{\text{loge(triglycerides)}} + 0.139*{\text{BMI}} + 0.718*{\text{loge(GGT)}} + 0.053*{\text{waist circumference}} - 15.745}} )/ (1 + e^{{0.953*{\text{loge(triglycerides)}} + 0.139*{\text{BMI}} + 0.718*{\text{loge (GGT)}} + 0.053*{\text{waist}}+ {\text{circumference}} - 15.745}} ) \times 100.$$

With parameters derived from the OGTT, homeostasis model assessment for insulin resistance (HOMA2-IR), Disposition Index and Insulinogenic Index were calculated. For HOMA2-IR, the computer program from Oxford University was used.[26, 27]. The Insulinogenic Index is calculated by dividing Δ_insulin_ by the 30-min glucose level (ΔI_0–30_/ΔG_0–30)_) [28]. Finally, the oral Disposition Index is the product of the IGI and 1/fasting insulin (ΔI_0–30_/ΔG_0–30_ × 1/fasting insulin) [29]. All samples were analysed in our central laboratory (ISO15189 certified) at the General Hospital in Vienna/ AKH Wien. Details on the used laboratory methods are available on the homepage of the institute of laboratory medicine, www.kilm.at. Data on breastfeeding at the postpartum visit was collected after the study via telephone visits and chart review.

### Assay

Gremlin-1 was analysed with a Gremlin 1 ELISA kit (MBS2020142) from My BioSource (https://biocheminfo.org/mbs2020142-gremlin-1-grem1-elisa-kit/). The detection range of this kit is 0.625–40 ng/mL with an inter-assay CV of < 12% and an intra-assay CV of < 10%. Sample collection, storage (− 20 °C), preparation of the standard curve and the analysis of the samples was done according to the manufacturer’s recommendations. We additionally used 2 internal control samples for each assay in order to check reproducibility of the results, which resulted in a calculated CV % at 9.2% aligned with the range given by the manufacturer. Gremlin-1 was measured at the visit < 20th GW, GW 24–28 and at around 12–14 weeks postpartum.

### Statistical analysis

As a first step, a descriptive data analysis including a Kolmogorov–Smirnov test and histograms was performed for all parameters. All non-parametrically distributed parameters were log-transformed. For the baseline characteristics table, continuous variables were summarized by mean ± SD and categorical variables by counts. Paired samples T tests were calculated to evaluate potential changes of the parameter Gremlin-1 during pregnancy and postpartum. To investigate whether Gremlin-1 levels differ between women with a normal glucose tolerance (NGT) and gestational diabetes mellitus (GDM), a univariate analysis of covariance (ANCOVA) at gestational week (GW 24–28) corrected for week of gestation was used. Gremlin-1 levels and parameters that had been described to potentially be related to Gremlin-1 in previous studies were tested with Pearson's correlation tests. Pairwise deletion was used for cases with missing records. As this is a post hoc analysis, a power analysis was omitted. For the statistical analysis, SPSS 25.0 (SPSS Inc, Chicago, USA) was used. A two-sided *p*-value < 0.05 was considered statistically significant.

## Results

### Baseline characteristics

In Table 1, the baseline characteristics of the study population at GW < 20, GW 24–28 and 12–14 weeks postpartum are described. At the baseline visit, the study participants were on average in gestational week 15 (± 2), were 34 (± 5) years old and had a BMI of 32.6 (± 6.7) kg/m^2^. Weight (GW < 20: 89.4 ± 19.9 kg, GW 24–28: 92.2 ± 18.5 kg) and waist circumference (GW < 20: 108 ± 16 cm, GW 24–28: 113 ± 13 cm) rose during pregnancy and dropped again at the postpartum visit (weight: 88.5 ± 19.5 kg, waist circumference: 106 ± 13 cm). Parameters of insulin resistance such as fasting insulin (GW < 20: 12.7 ± 8.5 µIU/mL, GW 24–28: 18.3 ± 17.5 µIU/mL, Postpartum: 10.9 ± 6.4 µIU/mL) and HOMA2-IR (GW < 20: 1.6 (± 1.05), GW 24–28: 2.12 (± 1.63), Postpartum 1.41 (± 0.83)) increased over the course of pregnancy and decreased postpartum. For more information, see Table 1.

**Table 1:** Baseline characteristics and metabolic parameters of visit at < 20 GW, GW 24–28 and at 12–14 weeks postpartum

N = 58	Baseline visit (< 20 GW)	GW 24–28	Postpartum
Gremlin-1 (ng/mL)	9.2 (± 8.4)	6.7 (± 5.7)	10.7 (± 13.1)
Age (years)	34 (± 5)	34 (± 5)	35 (± 5)
Week of gestation/ weeks postpartum	15 (± 2)	26 (± 1)	13 (± 6)
BMI (kg/m^2^)	32.6 (± 6.7)	33.6 (± 6.2)	32.5 (± 6.5)
Weight (kg)	89.4 (± 19.9)	92.2 (± 18.5)	88.5 (± 19.5)
Waist (cm)	108 (± 16)	113 (± 13)	106 (± 13)
Hip (cm)	117 (± 14)	118 (± 12)	117 (± 14)
Blood pressure systolic (mmHg)	116 (± 13)	115 (± 11)	114 (± 12)
Blood pressure diastolic (mmHg)	72 (± 10)	70 (± 9)	74 (± 9)
Heart rate (bpm)	80 (± 10)	86 (± 12)	77 (± 10)
Oestradiol (pg/mL)	3921 (± 3013)	11,258 (± 3943)	61 (± 70)
Testosterone (ng/mL)	0.67 (± 0.41)	0.63 (± 0.36)	0.25 (± 0.17)
Progesterone (ng/mL)	31.25 (± 11.56)	61.78 (± 19.49)	1.20 (± 2.48)
Fasting glucose (mg/dl)	84 (± 9)	84 (± 12)	85 (± 9)
Fasting insulin (µIU/mL)	12.7 (± 8.5)	18.3 (± 17.5)	10.9 (± 6.4)
HOMA2-IR	1.6 (± 1.05)	2.12 (± 1.63)	1.41 (± 0.83)
Disposition Index	7.12 (± 9.16)	5.03 (± 3.48)	8.64 (± 13.50)
Insulinogenic Index	1.68 (± 1.91)	1.58 (± 0.90)	1.55 (± 1.57)
HbA1c (in %)	5.1 (± 0.3)	4.9 (± 0.3)	5.2 (± 0.3)
Cholesterol (in mg/dL)	189 (± 29)	225 (± 36)	189 (± 35)
LDL-C (in mg/dL)	96 (± 31)	114 (± 38)	110 (± 30)
HDL-C (in mg/dL)	65 (± 14)	73 (± 19)	59 (± 16)
TG (in mg/dL)	129 (± 51)	180 (± 53)	99 (± 50)
Fatty liver index	62.6 (± 28.8)	70.8 (± 25.0)	55.0 (± 30.0)
AP (in U/L)	58 (± 43)	76 (± 60)	88 (± 68)
GGT (in U/L)	30 (± 119)	16 (± 39)	43 (± 158)
AST (in U/L)	20 (± 8)	19 (± 9)	26 (± 10)
ALT (in U/L)	22 (± 15)	18 (± 13)	34 (± 22)
Creatinine (in mg/dL)	0.53 (± 0.08)	0.51 (± 0.07)	0.70 (± 0.10)
Osteocalcin (in ng/ml)	12.9 (± 4.2)	15.6 (± 13.2)	26.9 (± 10.5)
P1NP (in µg/L)	42 (± 15)	47 (± 19)	70 (± 25)
CRP (in mg/dL)	2.20 (± 0.09)	2.16 (± 0.09)	2.31 (± 0.10)

Continuous variables were summarized by mean ± standard deviation (SD)

*BMI* Body mass index, *CRP* C-reactive protein, *HOMA2-IR* homeostasis model assessment for insulin resistance, *HbA1c* haemoglobin A1c, *LDL_C* low density lipoprotein cholesterol, *HDL-C* high density lipoprotein cholesterol, *TG* triglycerides, *AP* alkaline phosphatase, *GGT* gamma-glutamyl transferase, *AST* aspartate aminotransferase, *ALT* alanine aminotransferase, *P1NP* procollagen type 1 N-terminal propeptide

Of all participating women, 19% (11/58 women) had a normal BMI, 26% (15/58 women) a BMI between 25 and 30 kg/m^2^, 31% (18/58 women) a BMI between 30 and 35 kg/m^2^ and 24% (14/58 women) a BMI of over 35 kg/m^2^ before pregnancy.

74% (35/47) of all participating women breastfed their child either exclusively or mostly at the time of the postpartum visit. 26% (12/47) had either not started breastfeeding or stopped within a week of the birth. 11 women could not be reached to provide data on breastfeeding.

### Gremlin-1 levels throughout pregnancy

**Fig. 1:**
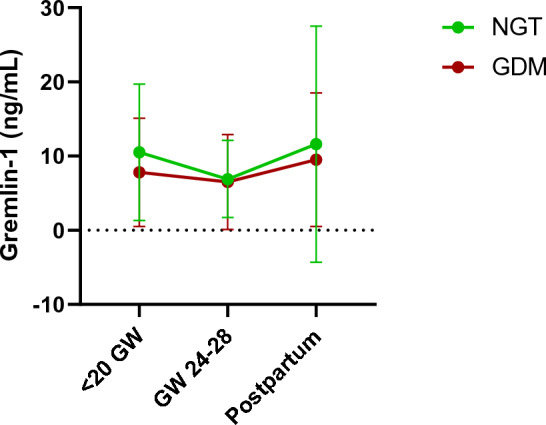
Gremlin-1 levels throughout pregnancy. Gremlin-1 levels decrease from < 20 GW (mean = 9.2, SD = 8.4) to GW 24 to 28 (mean = 6.7, SD = 5.7 pg/ml, *p* = 0.033) and rise again postpartum (mean = 10.7, SD = 13.1, *p* = 0.339). Women with NGT (green, N = 29) had slightly higher levels of Gremlin-1 than women with GDM (red, N = 29) during pregnancy (for both visits: mean^NGT^ = 9.1 ± 5.8, mean^GDM^ = 5.9 ± 3.1; *p* = 0.128) and postpartum (mean^NGT^ = 11.6 ± 15.9, mean^GDM^ = 9.5 ± 9.0; *p* = 0.807), however, not significantly higher. GW = gestational week, NGT = normal glucose tolerance, GDM = gestational diabetes mellitus

In all women, Gremlin-1 levels were significantly higher at < 20 GW (mean = 9.2 ng/mL, SD = 8.4 ng/mL) compared to GW 24–28 (mean = 6.7 ng/mL, SD = 5.7 ng/ml, *p* = 0.033), for details see Fig. 1. Gremlin-1 levels rose again postpartum (mean = 10.7 ng/mL, SD = 13.1 ng/mL, *p* = 0.339), albeit not significantly. When split into NGT (29 women) and GDM (29 women), Gremlin-1 levels of women with NGT are not significantly higher than those of women with GDM, neither during pregnancy (mean^NGT^ = 9.1 ± 5.8 ng/mL, mean^GDM^ = 5.9 ± 3.1 ng/mL; *p* = 0.128) nor at the postpartum visit (mean^NGT^ = 11.6 ± 15.9 ng/mL, mean^GDM^ = 9.5 ± 9.0 ng/mL; *p* = 0.807).

Furthermore, a univariate ANCOVA correcting for week of gestation resulted in no significant differences (corrected mean^LOG10(NGT)^ = 0.704 ± 0.085, mean^LOG10(GDM)^ = 0.603 ± 0.088; *p* = 0.239) in Gremlin-1 levels between NGT and GDM at GW 24–28.

When split into different BMI categories, Gremlin-1 levels did not display any differences (< 30 kg/m^2^: mean = 0.74 ± 0.35 compared to > 30 kg/m^2^: mean = 0.85 ± 0.47, *p* = 0.372).

At the first visit during pregnancy, Gremlin-1 correlated negatively with the IGI (r_p_ = − 0.324, *p* = 0.044) and osteocalcin (r_p_ = − 0.318, *p* = 0.026). At GW 24–28, Gremlin-1 was negatively associated with P1NP (r_p_ = − 0.334, *p* = 0.027) and waist-hip ratio (WHR) (r_p_ = − 0.300, *p* = 0.043). Postpartum, Gremlin-1 was related to weight (r_p_ = 0.305, *p* = 0.046), hip circumference (r_p_ = 0.324, *p* = 0.030), waist-hip ratio (r_p_ = − 0.317, *p* = 0.034), diastolic blood pressure (r_p_ = 0.301, *p* = 0.047) and the FLI (r_p_ = 0.424, *p* = 0.004), whereas it correlated negatively with osteocalcin (r_p_ = − 0.436, *p* = 0.005). For details, see Table 2.

**Table 2:** Pearson's correlation analysis of Gremlin-1 levels during pregnancy at GW < 20, GW 24–28, 12–14 weeks postpartum as well as the difference in Gremlin-1 levels (Gremlin-1_diff) correlated with the change in parameters (parameter_diff) from GW < 20 to GW 24–28

Pearson' correlation	GW < 20		GW 24–28		Gremlin-1_diff correlated with parameter_diff		PP	
	r_p_	*p*	r_p_	*p*	r_p_	*p*	r_p_	*p*
Age (years)	− 0.177	0.220	0.179	0.336	− 0.007	0.966	− 0.006	0.975
Weight (kg)	0.058	0.689	0.063	0.670	− 0.046	0.944	**0.305**	**0.046**
BMI	0.026	0.860	− 0.026	0.861	− 0.041	0.796	0.297	0.056
Waist (cm)	− 0.006	0.968	− 0.060	0.688	− 0.046	0.772	0.179	0.240
Hip (cm)	0.073	0.615	0.117	0.440	− 0.035	0.829	**0.324**	**0.030**
Waist-hip ratio (cm)	− 0.129	0.373	** − 0.300**	**0.043**	− 0.053	0.740	** − 0.317**	**0.034**
Blood pressure systolic (in mmHg)	0.033	0.820	0.070	0.638	− 0.187	0.230	0.236	0.124
Blood pressure diastolic (in mmHg)	− 0.078	0.591	− 0.166	0.259	− 0.181	0.245	**0.301**	**0.047**
Heart rate (bpm)	0.050	0.728	− 0.012	0.933	− 0.178	0.254	0.200	0.194
Fasting insulin (in µIU/mL)	0.018	0.911	− 0.096	0.567	− 0.336	0.069	0.131	0.415
Fasting glucose (in mg/dl)	0.040	0.781	− 0.125	0.432	− 0.242	0.149	− 0.071	0.649
HOMA2-IR	0.053	0.735	− 0.063	0.709	− 0.240	0.202	0.153	0.334
Disposition Index	− 0.278	0.091	0.269	0.150	** − 0.479**	**0.018**	0.144	0.375
Insulinogenic Index	** − 0.324**	**0.044**	0.097	0.609	− 0.379	0.061	0.245	0.128
HbA1c (in %)	− 0.127	0.381	− 0.004	0.977	–	–	− 0.138	0.372
Cholesterol (in mg/dL)	0.107	0.463	0.014	0.924	− 0.296	0.057	0.229	0.136
LDL-C (in mg/dL)	0.019	0.898	0.236	0.106	0.154	0.330	0.232	0.130
TG (in mg/dL)	0.193	0.184	− 0.060	0.688	− 0.192	0.224	0.118	0.445
Oestradiol (in pg/mL)	0.089	0.549	0.071	0.630	− 0.085	0.596	0.031	0.845
Testosterone (in pg/mL)	0.018	0.902	0.010	0.944	− 0.091	0.576	0.087	0.583
Osteocalcin (in ng/mL)	** − 0.318**	**0.026**	− 0.033	0.822	0.182	0.248	** − 0.436**	**0.005**
P1NP (in µg/L)	− 0.254	0.096	** − 0.334**	**0.027**	− 0.290	0.096	− 0.145	0.413
Creatinine (in mg/dL)	− 0.189	0.194	0.083	0.573	− 0.217	0.168	− 0.165	0.284
Fatty liver index	0.100	0.492	− 0.020	0.894	− 0.183	0.246	**0.424**	**0.004**
GGT (in U/L)	0.191	0.189	− 0.114	0.441	0.299	0.055	0.130	0.399
AST (in U/L)	0.260	0.072	0.012	0.937	− 0.048	0.763	0.046	0.764
ALT (in U/L)	0.068	0.642	− 0.079	0.596	− 0.122	0.441	0.024	0.878
CRP (mg/dL)	− 0.111	0.447	0.001	0.994	0.045	0.778	0.072	0.642

Significance level is *p* < 0.05

*GW* gestational week, *BMI* Body mass index, *CRP* C-reactive protein, *HOMA2-IR* homeostasis model assessment for insulin resistance, *HbA1c* haemoglobin A1c, *LDL-C* low density lipoprotein cholesterol, *HDL* high density lipoprotein, *TG* triglycerides, *AP* alkaline phosphatase, *GGT* gamma-glutamyl transferase, *AST* aspartate aminotransferase, *ALT* alanine aminotransferase, *P1NP* procollagen type 1 N-terminal propeptide

As for the change of Gremlin-1 levels during pregnancy, Gremlin-1 levels correlated negatively with the Disposition Index (r_p_ = − 0.479, *p* = 0.018) and the Insulinogenic Index (r_p_ = − 0.479), albeit with a significance level of *p* = 0.061.

**Fig. 2:**
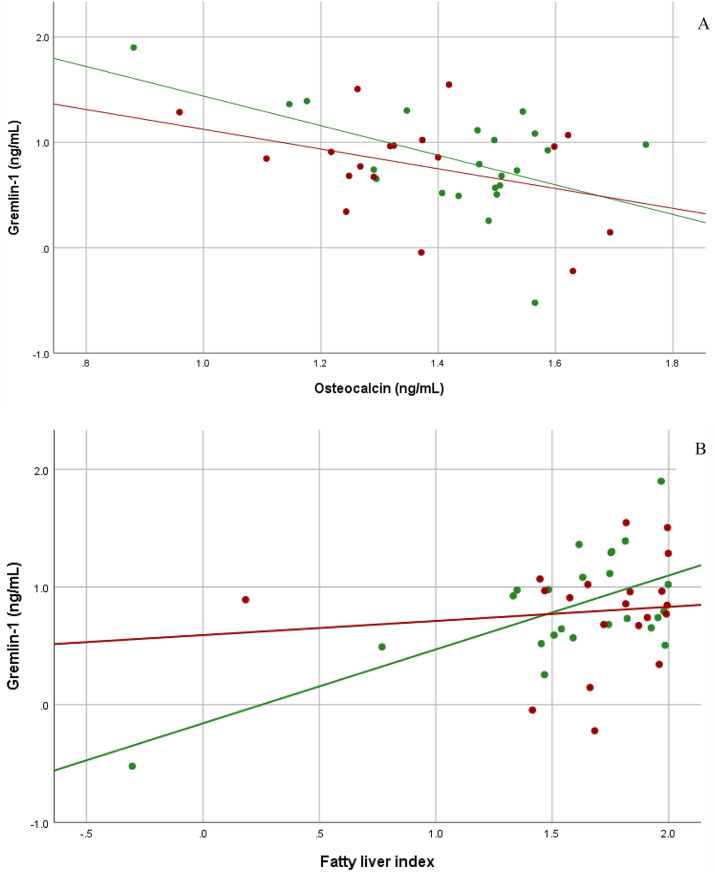
**A** &** B** Grouped scatter dot plot according to glucose tolerance (green = NGT, red = GDM) depicting the correlation of Gremlin-1 (log-transformed) with osteocalcin levels (2A) and the fatty liver index (2B) at the postpartum visit. Gremlin-1 levels correlated negatively with osteocalcin levels in women with NGT (r_p_ = − 0.527, *p* = 0.012) and GDM (r_p_ = − 0.369, *p* = 0.132). In women with NGT, Gremlin-1 correlated positively with the fatty liver index (r_p_ = 0.651, *p* = 0.001). Gremlin-1 levels in women with GDM display the same trend, albeit not statistically significant (r_p_ = 0.107, *p* = 0.654). GW = gestational week, NGT = normal glucose tolerance, GDM = gestational diabetes mellitus

When split into GDM and NGT, Gremlin-1 correlated negatively with age (r_p_ = − 0.509, *p* = 0.008), the Disposition Index (r_p_ = − 0.572, *p* = 0.007) and the Insulinogenic Index (r_p_ = − 0.599, *p* = 0.003) in women with NGT at GW < 20, but positively with the Insulinogenic Index (r_p_ = 0.524, *p* = 0.031) in women with GDM at GW < 20. Gremlin-1 was related to GGT (r_p_ = 0.600, *p* = 0.002) and ALT (r_p_ = 0.453, *p* = 0.026) in women with GDM at GW < 20. Postpartum, Gremlin-1 levels were positively associated with cholesterol (r_p_ = 0.576, *p* = 0.008) and LDL-C levels (r_p_ = 0.518, *p* = 0.019) in women with GDM. In women with NGT, we found a positive relation of Gremlin-1 levels to systolic (r_p_ = 0.509, *p* = 0.013) and diastolic blood pressure (r_p_ = 0.515, *p* = 0.012) as well as heart rate (r_p_ = 0.487, *p* = 0.018) and the fatty liver index (r_p_ = 0.651, *p* = 0.001), but a negative correlation to osteocalcin levels (r_p_ = − 0.527, *p* = 0.012) at the postpartum visit. For details see Fig. 2A and B.

Gremlin-1 levels were not statistically different between women who breastfed their child and those who were not able to breastfeed at the postpartum visit (breastfeeding: mean = 9.1 ± 5.8 ng/mL, not breastfeeding: mean = 5.9 ± 3.1 ng/mL; *p* = 0.128).

## Discussion

Up to this point, there has been no evaluation of Gremlin-1 in pregnancy, postpartum or in relation to gestational diabetes mellitus status. In the present study, Gremlin-1 decreased from < 20 GW to GW 24–28. Although patients with type 2 diabetes mellitus have shown higher serum levels of Gremlin-1 than controls with a normal glucose tolerance [4], Gremlin-1 levels were not significantly different between patients with GDM compared to NGT. Furthermore, Gremlin-1 levels were negatively related to the bone formation markers osteocalcin and P1NP in pregnancy and postpartum.

Although Gremlin-1 expression has been described as increased in obesity in general, levels are described as the highest in patients with type 2 diabetes mellitus in previously published studies [4]. However, in our study, both pregnant women with GDM and NGT were mostly overweight or obese and all women increasingly insulin resistant due to physiological, pregnancy-related changes in insulin secretion and resistance [13, 14]. Gremlin-1 and 2 have been demonstrated to be related to both obesity and insulin resistance [4, 30, 31], which might explain the lack of differences in Gremlin-1 levels between NGT and GDM in our population. GDM status, in contrast to patients with type 2 diabetes mellitus, might, thus, not be a determining factor for Gremlin-1 levels during pregnancy. Another explanation would be the high standard deviation of Gremlin-1 in women with NGT which might be due to a higher metabolic flexibility to adapt to changes in insulin and glucose metabolism in pregnancy.

Moreover, we saw a tendency of Gremlin-1 levels being higher in NGT than GDM, which is not in line with results on type 2 diabetes mellitus patients outside of pregnancy [4]. In pregnancy, increasing insulin production and resistance is a physiological process to divert glucose to the placenta and, thus, the foetus [13, 14]. Hedjazifar et al. demonstrated that recombinant Gremlin-1 inhibits glucose uptake in response to insulin, whereas anti-Gremlin antibodies have a sensitizing effect on insulin-induced glucose uptake [4]. In contrast to Hedjazifar et al. and other studies where Gremlin-1 has been shown to be elevated in states of higher insulin resistance [4, 30, 31], we saw no positive association of Gremlin-1 levels with markers of insulin resistance in our study. Rather, Gremlin-1 correlated negatively with the Insulinogenic Index, a marker of beta cell insulin secretion, and the Disposition Index, an integrated measure of beta cell function during pregnancy. Furthermore, Gremlin-1 levels dropped significantly during pregnancy, a period of both rising insulin resistance and secretion [13, 14]. Thus, Gremlin-1 levels might be down-regulated in pregnancy in order to facilitate higher levels of insulin secretion and resistance and deflect nutritional resources such as glucose towards the foetus, especially in progressing pregnancy. Or the fact that most participating women were overweight or obese attenuated the effect of insulin resistance on Gremlin-1 levels in pregnancy. However, Gremlin-1 levels were not different between women in different BMI categories in pregnancy or postpartum in our analysis. Thus, the full extent of the role of Gremlin-1 in insulin secretion and resistance during pregnancy is yet to be determined. Moreover, when split into GDM and NGT, Gremlin-1 correlated negatively with the Disposition Index and the Insulinogenic Index in women with NGT, but positively with the Insulinogenic Index in women with GDM at GW < 20. At this point, we are not able to explain this discrepancy.

Postpartum, Gremlin was related to weight, hip circumference, diastolic blood pressure and the fatty liver index (FLI), whereas it correlated negatively with WHR. A positive relation to the FLI is in line with previously published literature stating higher levels of Gremlin-1 in patients with NAFLD or NASH [4]. In contrast to our result, Koroglu et al. previously described a positive correlation of waist to hip ratio (WHR) with Gremlin-1 levels [31]. However, the relation of WHR to Gremlin-1 levels might be distorted in pregnant women due to the growing abdominal circumference during gestation. As to why the negative correlation persists in the postpartum visit, we are not able to offer an explanation at this point due to the exploratory character of this study.

To the best of our knowledge, no study investigating sex hormones and Gremlin-1 has been published to this day. In our investigation, Gremlin-1 levels were negatively related to progesterone levels measured postpartum. A previous publication described a negative correlation between weight and progesterone levels [32]. As Gremlin-1 levels have shown to be positively related to weight [4, 30, 31], this might indirectly explain our findings. However, more investigations are necessary to explain a potential connection between Gremlin-1 and sex hormone levels. Interestingly, in pregnancy, no associations of Gremlin-1 with sex hormones were found. These contradictory results need further investigation in future studies.

During pregnancy and postpartum, Gremlin correlated negatively with osteocalcin and P1NP both peptides involved in the stimulation of bone formation processes. This negative association corresponds to Gremlin-1 being a bone morphogenic protein expressed in osteoblasts that acts as an antagonist of osteoblastic differentiation [33], in mice, overexpressing Gremlin-1 led to osteopenia and spontaneous fractures [33]. In a pregnancy cohort, Winhofer et al. described osteocalcin as being related to higher insulin secretion which aligns with our results of Gremlin-1 correlating negatively with the IGI, a marker of insulin secretion [34].

A limitation of this study is that our study population is a selected group of women with a high risk for GDM due to the tertiary hospital setting and, thus, a greater proportion of women were overweight or obese and/or developed GDM. Other limitations include this study being monocentric with only Caucasian women, thus, only limited applicability to other ethnicities and/or regions. Furthermore, we were unable to reach 11 of 58 participating women to collect data on breastfeeding.

To conclude, this study offers a first insight into potential roles of Gremlin-1 during gestation with a focus on insulin secretion and resistance. Future studies including normal-weight women and pre-pregnancy Gremlin-1 levels might help to gather more information on the role of Gremlin-1 in pregnancy, insulin secretion and resistance as well as GDM status.

## Data Availability

The data presented in this study are available on request from the corresponding author.
